# Phospholipids of Animal and Marine Origin: Structure, Function, and Anti-Inflammatory Properties

**DOI:** 10.3390/molecules22111964

**Published:** 2017-11-14

**Authors:** Ronan Lordan, Alexandros Tsoupras, Ioannis Zabetakis

**Affiliations:** Department of Biological Sciences, University of Limerick, V94 T9PX Limerick, Ireland; Ronan.Lordan@ul.ie (R.L.); Alexandros.Tsoupras@ul.ie (A.T.)

**Keywords:** phospholipids, atherosclerosis, inflammation, anti-inflammatory, dairy, marine, meat, egg, nutrition

## Abstract

In this review paper, the latest literature on the functional properties of phospholipids in relation to inflammation and inflammation-related disorders has been critically appraised and evaluated. The paper is divided into three sections: Section 1 presents an overview of the relationship between structures and biological activities (pro-inflammatory or anti-inflammatory) of several phospholipids with respect to inflammation. Section 2 and Section 3 are dedicated to the structures, functions, compositions and anti-inflammatory properties of dietary phospholipids from animal and marine sources. Most of the dietary phospholipids of animal origin come from meat, egg and dairy products. To date, there is very limited work published on meat phospholipids, undoubtedly due to the negative perception that meat consumption is an unhealthy option because of its putative associations with several chronic diseases. These assumptions are addressed with respect to the phospholipid composition of meat products. Recent research trends indicate that dairy phospholipids possess anti-inflammatory properties, which has led to an increased interest into their molecular structures and reputed health benefits. Finally, the structural composition of phospholipids of marine origin is discussed. Extensive research has been published in relation to ω-3 polyunsaturated fatty acids (PUFAs) and inflammation, however this research has recently come under scrutiny and has proved to be unreliable and controversial in terms of the therapeutic effects of ω-3 PUFA, which are generally in the form of triglycerides and esters. Therefore, this review focuses on recent publications concerning marine phospholipids and their structural composition and related health benefits. Finally, the strong nutritional value of dietary phospholipids are highlighted with respect to marine and animal origin and avenues for future research are discussed.

## 1. Introduction

Lipids are a very heterogenic class of biomolecules with a wide range of structures and functions. Lipids can be divided into two major sub-classes, neutral lipids (such as Triacylglycerol’s (TAGs), waxes, and terpenes), which are molecules with long hydrophobic hydrocarbon chains lacking a free polar group, and polar lipids (such as phospholipids, glycolipids, etc.) that, apart from their hydrophobic hydrocarbon residues, also bare polar-hydrophilic group such as a carbohydrate-group, or a phosphate head group with a hydrophilic residue within their structure.

### 1.1. Phospholipid Classes and Biological Functions

**Figure 1:**
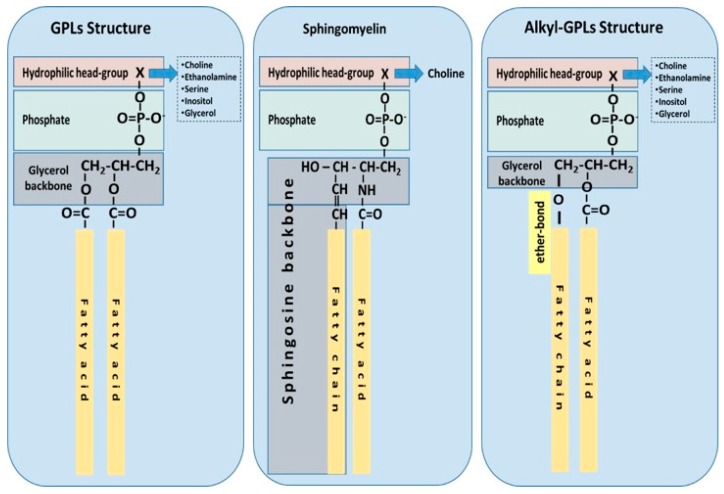
The most common structures of phospholipids are depicted: phospholipids with a glycerol backbone (GPLs); sphingomyelin as a representative of a sphingosine-backbone phospholipid (SPLs); and alkyl-phospholipids (Alkyl-GPLs) that have a fatty chain linked with an ether-bond at the *sn*-1 position of the glycerol backbone.

Ubiquitous to all tissues, phospholipids (PLs) are essential components of cell membranes consisting of a hydrophilic head group and a hydrophobic tail giving phospholipids their amphiphilic properties. Glycerophospholipids (GPLs) share a common structure consisting of two fatty acid (FA) molecules esterified in the *sn*-1 and *sn*-2 positions of the glycerol moiety. This portion of the molecule contributes to its hydrophobicity. The *sn*-3 position consists of a phosphate group with a hydrophilic residue that contributes hydrophilicity (Figure 1). The simplest GPL is phosphatidic acid (PA), others are named after the hydrophilic residue/group attached to the phosphate group. Four main groups have been identified: ethanolamine, inositol, serine, and choline. These groups form the most biologically important phospholipids, which are phosphatidylethanolamine (PE), phosphatidylinositol (PI), phosphatidylserine (PS) and phosphatidylcholine (PC). Lysophospholipids (Lyso-PLs) refer to phospholipids whose fatty acid chain has been removed from either the *sn*-1 or *sn*-2 position. Sphingolipids (SPLs) contain the long-chain amino alcohol sphingosine (instead of glycerol) esterified to a fatty acid and a phosphate group. Sphingomyelin (SM) is the most representative SPL, which consists of sphingosine and bares a choline molecule. SM is found in high quantities in brain and neural tissues membranes (Figure 1).

The biological importance of these PLs derives from their amphiphilic properties. The hydrophilic head and the hydrophobic tail create a lipid bi-layer that allows for the assembly of cell and organelle membranes [1,2,3]. These phospholipid-based bilayers form selectively permeable barriers, which are essential for effective separation of a cell or organelle from its surroundings. These properties allow for low membrane permeability for cellular constituents such as nutrients and ions, while the organisation into a lipid bilayer provides the perfect matrix in which the membrane-integral proteins are embedded. No mammalian membranes or cells are formed without PLs and the integrity and function of the external (cellular) and internal (subcellular) membrane systems depends on their composition and on the integrity of their phospholipid structure. Besides GPLs and SPLs, biological membranes are also made up of glycolipids and cholesterol, as well as of integral and peripheral membrane proteins.

Other forms of GPLs exist, which differ from the general structure of GPLs, such as ether-linked GPLs that bare other hydrocarbon chains (saturated or unsaturated, or with hydroxyl-groups, etc.) ether-linked to the *sn*-1 position of the glycerophosphate backbone, instead of a fatty acid bound by ester bonds to the *sn*-1 position of the glycerol backbone (Figure 1). Ether-linked GPLs can be found as minor constituents of cell membranes in both prokaryotes and eukaryotes, but they are abundant in archaeal organisms [4]. Some exist as bioactive molecules that seem to be maintained through evolution from archaeal to eukaryotic organisms because of their lipid signalling bioactivities, especially in eukaryotic organisms. One such examples includes plasmalogens and platelet-activating factor, also known as PAF (1-*O*-alkyl-2-acetyl-*sn*-glyceryl-3-phosphorylcholine) [5], which is potent inflammatory mediator involved in the innate immune response and chronic inflammatory diseases [6,7].

The lipid composition of biological membranes represents a taxonomic signature that distinguishes the different kingdoms of life. Differences in ester and/or ether bonded fatty acid chains at the glycerol backbone exist between different kinds of organisms [4]; in addition, the fatty acid composition of PLs varies depending on their origin [8]. Due to their amphipathic properties, naturally occurring PLs either from plant or animal origin, generally contain an unsaturated fatty acid in the *sn*-2 position, such as oleic acid, linoleic acid, α-linolenic acid, arachidonic acid (pro-inflammatory molecule usually from animal origin) or eicosapentaenoic acid (anti-inflammatory molecule usually from marine origin), whereas the *sn*-1 position predominantly carries a saturated fatty acid (SFA), such as stearic acid or palmitic acid [9]. The correct ratio of saturated to unsaturated fatty acids in the phospholipid membrane is essential to sustain the membrane characteristics, since the fatty acid composition and degree of saturation directly affects the fluidity of the cell membrane. Equally, the correct ratio can have a significant effect on cellular processes such as the formation of lipid rafts. Lipid rafts are dynamic membrane micro-domains with a high content of cholesterol and PLs predominantly carrying SFA, which are implicated in apoptosis, cellular proliferation, and unsaturated fatty acids that act as precursors for the synthesis of pro-inflammatory mediators called eicosanoids (prostaglandins (PGs), thromboxanes (TX), leukotrienes (LT), and lipoxins (LX)) [10,11]. 

Even though the main function of PLs is to support the formation and biofunctionality of cell membranes, there are specific varied PLs that perform specialised functions in the subcellular micelles and organelles. For example, PLs are structural and functional constituents of the surface monolayers of lipoproteins (which transport lipids to tissues via the blood stream), the pleura and alveoli of the lung and are constituents of the pericardium, joints, peritoneal and gastrointestinal surfactants, while together with cholesterol and bile acids they form mixed micelles in the gallbladder for fat emulsification [12]. In addition, some PLs act as lipid mediators of inflammation that have the ability to influence immunological processes at the cellular level (i.e., PAF) [7]. PLs also contain bound PUFAs to be released on demand as precursors of prostaglandins and other eicosanoids [11], while other PLs and their metabolites are a source of secondary messengers in cell signalling (e.g., diacylglycerols, phosphoinositides, etc.) [13], and carry out essential functions within organelles such as the mitochondria [14]. Therefore, not only are PLs integral structural lipids in cell membrane formation, function and integrity, but research has also identified that they possess a plethora of additional functions in various cell types and organisms, which will be discussed further in this review.

### 1.2. Glycerophospholipid and Sphingophospholipid Biosynthesis

In mammalian cells, GPL synthesis requires a diacylglycerol unit, which is provided by either diacylglycerol or CDP-diacylglycerol. The generation of these precursors initiates through the enzyme glycerol-3-phosphate acyltransferase (situated in the external leaflet of the mitochondrial membrane and of the endoplasmic reticulum), which links a fatty acid-CoA (generally a SFA) to the *sn*-1 position of glycerol-3-phosphate to generate lyso-PA. Acylglycerol-3-acyltransferase is required for the subsequent formation of PA in the endoplasmic reticulum, whereby it esterifies another fatty acid-CoA (generally an unsaturated FA) to the *sn*-2 position of glycerol. PA then becomes the substrate for two significant metabolic enzymatic pathways. The first pathway is controlled by a cytosolic phosphatidic acid phosphatase enzyme, which takes place in the membrane of the endoplasmic reticulum and produces diacylglycerols (DAG) by removing the phosphate group from the *sn*-3 position of PA. Triacylglycerols (TAG) are formed by the esterification of another fatty acid to the *sn*-3 position; these then become the main energy source in the body. Alternatively, CDP-diacylglycerol synthase, an enzyme associated primarily with the endoplasmic reticulum, catalyses a reaction between CTP and PA leading to the formation CDP-diacylglycerol. In the second pathway for PA synthesis, dihydroxyacetone-P is acylated to 1-acyl-dihydroxyacetone-P, which is subsequently converted to lyso-PA and then PA [1,15,16].

The synthesis of PC and PE occurs in the cytosol following the enzymatic addition of either a choline or ethanolamine to PA [17]. The biosynthesis of PS requires the presence of PC and PE. In terms of PE, PS synthesis occurs in the endoplasmic reticulum through two metabolic pathways, which use differential enzymes and substrates. Initially PC exchanges a choline with a serine molecule in the presence of PS synthase I, leading to the final products of PS and choline. Synthesis of PS from PE follows a similar pathway where PS synthases II catalyses the substitution of an ethanolamine head for a serine head, leading to the final products of PS and ethanolamine. In the presence of the same enzymes, the latter reaction is unique, as it is reversible, thus PS can release serine and replace it with ethanolamine [1,18]. PI is also biosynthesised in the endoplasmic reticulum where CDP-diacylglycerol binds to inositol, by the enzymatic actions of CDP-diacylglycerol phosphatidyl transferase. These reactions result in the production of PI and cytidine monophosphate (CMP). Other essential molecules often associated with the polar fraction of lipids such as cardiolipin (CL) are produced through the same pathway [1,19].

The synthesis of sphingomyelin starts in the endoplasmic reticulum and after a series of enzymatic reactions finishes in the Golgi apparatus and the plasma membrane. Synthesis begins with the condensation of serine and palmitoyl CoA by serine palmitoyltransferase forming 3-ketosphinganine, which is then reduced to dihydrosphingosine that is then *N*-acylated by one of six ceramide synthases (CerS1–CerS6), each using specific acyl chains, generally with a SFA or MUFA with 16–26 carbons, forming dihydroceramides that are subsequently dehydrogenated to ceramides by dihydroceramide desaturase. The reaction is catalysed by the enzymes sphingomyelin synthase I and sphingomyelin synthase II, which produces SM and diacylglycerols from the substrates ceramide and PC [1,20].

Plasmalogens are mainly synthesised in peroxisomes. They contain an aliphatic hydrocarbon chain at the *sn*-1 position of the glycerol linked via vinyl-ether binding derived from PC and PE. Generally plasmalogens are esterified with highly unsaturated fatty acids such as docosahexaenoyl or arachidonoyl fatty acid at the *sn*-2 position of glycerol [21]. The functions of plasmalogens are not yet fully understood, however it is proposed that they may act as potential biomarkers for age related diseases, oxidative stress and systemic inflammation [22].

### 1.3. Inflammation and Lipid Inflammatory Mediators

Inflammation is a necessary protective response of the innate immune system in response to physiological triggers such as pathogens or damaged cells, whereby the tissue is repaired, or the pathogenic insult is eliminated. However, excessive inflammation can lead to tissue injury [23]. Diet and lifestyle are a key modifiable risk factor for the prevention of chronic diseases. It has been established that a maladaptive diet is one of the dominant underlying causes of systemic inflammation through exaggerated postprandial elevations in plasma glucose and triglycerides. Due to the increased intake of heavily processed foods with high calorific value, postprandial hyperlipaemia and hyperglycaemia are common, postprandial lipaemia is an independent risk factor for cardiovascular disease (CVDs), obesity, metabolic syndrome and type II diabetes. The production of excess plasma reactive oxygen species (ROS) occurs due to the increased levels of postprandial glucose and triglycerides, which can lead to a pro-inflammatory state [23,24,25,26]. Activated immune cells are essential in preventing long lasting damage to the host, as they can maintain or resolve the inflammatory response. If an inflammatory response is not resolved the subsequent inflammatory microenvironment will disrupt tissue homeostasis leading to a systemic inflammatory condition. Several conditions owe their onset and progression to systemic inflammation including cancer, kidney disorders, obesity, type II diabetes mellitus, atherosclerosis and various CVDs. For further reading on the typical inflammatory response, see the comprehensive review of Medzhitov [27] and the works of Demopoulos et al. [28] and Libby et al. [29].

The initiation and resolution of the inflammatory response involves the complex and coordinated expression of many factors, including cytokines like the Interleukin-1 (IL-1) family, Interleukin-6 (IL-6), Tumour Necrosis Factor-α (TNF-α), Interferon-γ (INFγ), chemokines, growth factors (vascular endothelial growth factor or VEGF), proteases, ROS, oxidised phospholipids (Ox-PLs) and lipid-mediators such as eicosanoids and PAF. These inflammatory signals induce a myriad of physiological processes, ranging from local vascular responses to systematic responses affecting the whole organism [30]. These molecules sustain the inflammatory process until the insult has been resolved. Under all conditions, chronic inflammation leads to a disturbed homeostasis, spiralling the physiological and immunological conditions towards a pro-inflammatory harmful setting involving cells and secreted factors [7]. Persistent induction and dysregulation of inflammation has been recognised as an integral feature of the pathology of several chronic conditions including CVDs, type II diabetes, obesity, renal disorders, cancer and Alzheimer’s disease [7,29,31,32,33,34,35].

Interestingly, in such pathological conditions common junctions of inflammatory cross-talk between several inflammatory signalling pathways exist and can lead to comorbidities in such diseases. Patients with a chronic inflammatory disease are at risk of developing other inflammatory conditions and vice versa, a chronic inflammatory condition can be a major risk factor for the development of a chronic inflammatory disease. For example, chronic inflammation observed in diabetic patients is one of the leading causes of disease complications, which manifests in decreased kidney function, eye maladies, heart attacks and strokes [36]. In addition, the development of several autoimmune diseases characterised by an increased inflammatory status (i.e., increased levels of eicosanoids and cytokines) such as rheumatoid arthritis, can lead to the induction and co-development of CVDs [37]. Periodontal disease patients also exhibit a high risk of co-developing atherosclerosis and CVDs [38,39,40]. Similarly, HIV patients are at risk of persistent inflammation, which can lead to chronic inflammatory diseases such as atherosclerosis and CVDs [41]. Specific inflammatory biomolecules such as lipid inflammatory mediators (PAF, eicosanoids, etc.), cytokines/chemokines, growth factors, and adhesion molecules play similar roles as the main instigators of these manifestations in inflammation [7].

As chronic inflammation is responsible for many complications evident in different diseases, its diagnosis and treatment constitute an enormous challenge for medical practitioners. Importantly, many therapeutic treatments employed up to date have failed to produce a desirable effect, since a permissive immune environment is a prerequisite for their proper function. Nowadays, there is no doubt that chronic inflammation and associated immunosuppression pose a serious obstacle in the prognostic and the therapeutic area, as they both develop with no palpable clinical signs, often leading to unforeseeable complications and possible unresponsiveness to various therapies [42]. Apart from therapeutic interventions, long-term lifestyle measures such as healthy nutrition and exercise may provide preventive results or countermeasures towards inflammatory manifestations. The most known lipid pro-inflammatory mediators produced and implicated in inflammatory physiological responses are the eicosanoids and PAF. Both eicosanoid and PAF inflammatory pathways have been found to be promising targets in respect to dietary interventions, especially to those with foods containing bioactive PLs [43,44].

Eicosanoids are locally acting bioactive signalling lipids considered to be oxidized derivatives of 20-carbon fatty acids including a wide range of molecules such as prostaglandins (PGs), thromboxanes (TXs), leukotrienes (LTs) and lipoxins (LXs), which regulate a diverse set of homeostatic and inflammatory processes linked to numerous diseases [45]. The major substrate for eicosanoid synthesis is arachidonic acid (ARA, a lipid that usually is bonded at the *sn*-2 position of membrane glycerophospholipids), but also related PUFAs. Several agonists and receptors induce inflammatory processes and the subsequent cytokine “storm” that accompanies them initiates the release of ARA and related PUFAs, resulting in an eicosanoid storm [45]. Inflammatory stimuli trigger the activation of phospholipase A2 enzymes that release ARA from the *sn*-2 position of membrane phospholipids. ARA acts in turn as a substrate for several enzymes, such as cyclooxygenase (COX), cytochrome P450 enzymes and lipoxygenase (LOX) [46]. From this plethora of simultaneous biochemical reactions, various pro-inflammatory molecules are formed including PGs, TXs, LTs and LXs, which are well known mediators and regulators of inflammation [47]. 

Drugs that target eicosanoid pathways have been used for over a century; aspirin is the oldest of the numerous effective non-steroidal anti-inflammatory drugs (NSAIDs) that have been marketed. Systematic characterisation of prostaglandin and leukotriene structures, biosynthetic pathways, natural receptors and biological functions have resulted in the production of new drugs that target eicosanoids in order to treat common inflammatory symptoms including swelling and pain. However, chronic diseases such as arthritis and atherosclerosis are largely unaffected by the inhibition of eicosanoids [45]. In addition, side effects have been attributed to the blocking of COX-1 or COX-2 [45]. Low doses of aspirin are now commonly prescribed as cardioprotective agents, which limit thromboxane formation by COX-1 in platelets without inhibiting COX-2 mediated PGI2 formation by endothelial cells.

On the other hand, omega-3 (ω-3) fatty acid supplementation (i.e., from fish-oil) is also commonly prescribed for the treatment of various inflammatory ailments and for cardioprotection, due to their interactions with the eicosanoid pathways. The clearest evidence for this reasoning is the use of ω-3 fatty acids, such as ω-3 eicosapentaenoic acid (EPA) and docosahexaenoic acid (DHA). These fatty acids are abundant in fish and fish oils and they have the ability to inhibit arachidonic acid metabolism by COX-1 (but less so by COX-2), in a similar manner to low dose aspirin [45]. In addition, it is reported that EPA and DHA derived lipid mediators are less potent inducers of platelet aggregation in contrast to ARA-derived lipid mediators, which they displace, providing thus an agonistic effect towards ARA [48,49]. ω-3 fatty acids, such as EPA and DHA, seem to benefit multiple risk factors including blood pressure, blood vessel function, heart function, blood lipids, and they have antithrombotic, anti-inflammatory and anti-oxidative actions [50]. In addition to absolute amounts of ω-6 and ω-3 fatty acid intake, the ω-6/ω-3 ratio plays an important role in increasing the development of obesity via both ARA eicosanoid metabolites and hyperactivity of the cannabinoid system, which can be reversed with increased intake of EPA and DHA [51]. However, despite the overwhelming amount of evidence on the beneficial effects of ω-3 PUFAs on human health, some controversy remains since several systematic reviews and meta-analyses have illustrated that there is insufficient evidence for this notion, with many studies highlighting the lack of benefit and possible risks associated with the consumption of ω-3 PUFA supplements. Furthermore, some of these studies indicate that the beneficial effects of fish intake on cerebrovascular risk are likely to be mediated through the interplay of a wide range of nutrients abundant in fish [52,53,54,55,56]. However, within the last decade there is evidence that when ω-3 PUFAs (such as EPA and DHA) are combined to PLs, they are more efficiently incorporated into tissue membranes and at much lower doses than when these PUFAs are combined to TAGs. These PLs containing ω-3 PUFAs seem to provide beneficial effects towards inflammation related disorders through specific mechanisms and a plethora of bioactivities including their ability to modulate the eicosanoid pathway [43,57,58,59].

Other PLs also seem to contribute directly and/or indirectly to several inflammatory cascades and thus are involved in the onset, progression and often the remediation of inflammatory diseases. These PLs include plasmalogens, PAF, oxidised PLs and PL carriers of FA precursors of eicosanoids [7,11,60,61,62]. Several foods contain compounds that have well established modes of anti-inflammatory action, whose pleiotropic therapeutic effectiveness, and lack of toxicity ensures clinical safety. It is important to stress that in contrast to drug-induced minimisation of inflammation along with their subsequent side effects, dietary interventions using PLs seem to protect against inflammatory manifestations without any reported side effects thus far. It is thought that these PLs attenuate the levels of inflammation towards pre-inflammatory homeostatic baseline levels.

PLs found in food products such as meat, eggs, dairy, seafood and vegetable sources such as soybean are defined as dietary PLs. These dietary PLs are ingested as part of a normal diet, however in recent years, research has focused on the beneficial health effects of dietary PLs, and their anti-inflammatory activities against chronic diseases, thus PLs are also available as dietary supplements exhibiting pleiotropic beneficial effects towards inflammation related disorders. These food derived PLs can not only influence membrane-dependent cellular functions but they also possess anti-inflammatory, anti-oxidant, anti-fibrogenic, anti-apoptotic, membrane-protective, and lipid-regulating effects with a positive impact on several diseases, apparently without severe side effects [8,12]. Furthermore, dietary PLs can reduce the side effects of some drugs and they can influence the fatty acid composition of the hosts PLs. The main animal sources of phospholipids include eggs, milks, meats and marine phospholipids. Interestingly, marine phospholipids are much higher in PUFAs [43], which makes them a promising functional ingredient in foods. The oral application of such dietary PLs has the potential to cause defined alterations of the fatty acid composition of membrane PLs, and thus several cellular functions (according to cell-type), including cell signalling and transport, as well as the modulation of membrane bound enzymes that may lead to health benefits.

Apart from eicosanoids, PAF is another potent lipid inflammatory mediator with pleiotropic effects [63]. PAF is synthesized throughout the body by the specific stimulation of various cell types such as platelets, macrophages, monocytes, eosinophils, basophils, and endothelial cells. PAF is mostly produced in the blood, lungs, kidney, myocardium, brain, liver, skin, saliva, retina, uterus, and embryo [64,65]. The levels of PAF present in biological tissue are regulated by a balance of its biosynthetic and catabolic enzymatic pathways [7]. However, apart from its enzymatic biosynthetic pathways, PAF and PAF-like lipids that share similar structures and bioactivities, can also be produced through the oxidation of other lipids by ROS. The production of these PAF-like lipids occurs during inflammation and oxidative stress. PAFs can also stimulate the production of ROS and nitrogenous species during oxidative and nitrosative stress in inflammation-induced endothelial dysfunction and atherosclerosis [66]. PAF and PAF-like molecules act through their binding to a unique G-protein coupled seven transmembrane receptor, subsequently triggering multiple intracellular signalling pathways, depending on the target cell and PAF-levels in blood or tissue [67]. PAF, in general, plays a vital role in various physiological processes such as mediation of normal inflammatory responses, regulation of blood pressure, regulation of coagulation responses, foetal implantation, lung maturation, initiation of parturition, and exocrine gland functions.

PAF is produced and released in large quantities by inflammatory cells in response to specific stimuli, such as upstream regulators (IL-1, IL-6, TNF-α, Endothelin, and PAF itself) [7,66,68,69]. Increased PAF-levels at the site of inflammation can activate several cell-types through its receptor. This leads to the production of a broad spectrum of PAF-effects depending on the cell-type and tissue, which is achieved through various downstream mediators, enhancing the production and release of PAF itself and several other mediators of inflammation such as eicosanoids, TNF-α, IL-1α, IL-6, IL-8, growth factors, ROS and the expression of selectins and integrins in the membranes of activated cells [7,28,66,68,69]. The interconnected crosstalk between PAF, pro-inflammatory up-stream mediators that induce PAF-production, and PAF-induced downstream mediators seem to be interrelated during inflammatory manifestations. These pathways serve as one of the main junctions between many inflammatory cascades that ultimately lead to endothelium dysfunction and inflammation-related disorders such as atherosclerosis, CVDs and cancer [7,28,66].

The exploration of possible therapeutic approaches focus on the PAF/PAF-receptor interaction, thus inhibiting the exacerbation of the complex PAF inflammatory pathways. There are several agonists of synthetic and natural origin [23,70], which can competitively or noncompetitively displace PAF from its binding sites [71,72]. Even though specific PAF-antagonists have exhibited promising results, the most prominent beneficial effects have been derived from PL extracts of several foods. These food extracts exhibit anti-inflammatory and anti-oxidant activities through inhibiting PAF-activities and/or downregulating its levels by affecting/modulating the activities of key-metabolic enzymes of PAF (upregulation of PAF-catabolic enzymes activities and/or simultaneous downregulation of the basic PAF biosynthetic enzymes) *in vitro* and *in vivo*. The *in vitro* and *in vivo* beneficial effects of these dietary PLs are summarised in Table 1. 

### 1.4. Dietary Phospholipids: Digestion and Absorption

Dietary fat is mainly composed of TAG with PLs accounting for 3–6% of total fat intake [96]. The daily intake of PLs is not exactly known, however the daily intake of PC/day is estimated to be 2–8 grams [8]. TAGs and PLs are digested and absorbed in different ways in the small intestine. TAG requires emulsification by bile salts prior to absorption, while PLs can spontaneously form micelles that can be conveyed in an aqueous environment. In contrast to TAGs, PLs are not hydrolysed by lingual or gastric lipases but by other enzymes located in the small intestine. Thus, PLs are almost completely absorbed in the intestine. The most common PL present in the intestinal lumen is PC which is derived mostly from bile (10–20 g/day in humans) with the remainder coming from the diet, while other PLs, such as PE, PS, and PI, are present in much smaller amounts [58].

In the lumen, most of PLs are hydrolysed at the *sn*-2 position by pancreatic phospholipase A2 (pPLA2) and then absorbed by the enterocytes as free FAs and lyso-PLs. The fatty acid chain length and unsaturation number influences fat digestion, absorption, transport, and metabolism at cellular level. For instance, medium-chain fatty acids are better absorbed than long chain fatty acids because they can be dissolved in the aqueous phase and then be absorbed bound to albumin and transported to the liver directly by the portal vein [97]. Lyso-PLs and some free-FA are re-esterified to PLs (while some free FAs bind to TAGs) and enter the bloodstream incorporated into the surface layer of chylomicrons, whereas TAGs are incorporated into the core of chylomicrons. However, a small proportion will also incorporate into very low-density lipoproteins (VLDL). After the TAG-rich particles of the chylomicron are degraded, PLs such as PC can be taken up by the high-density lipoprotein (HDL) fraction, which occurs relatively rapidly, within 5–6 h of PLs ingestion [98,99]. Via HDL, PLs can be transferred into cells of numerous tissues and organs (e.g., liver, muscle, kidneys, lung, tumour cells, etc.) [43,100,101]. In contrast to GPLs, digestion of SM in the intestine is slow and incomplete, with initial hydrolysis of SM to ceramide by alkaline sphingomyelinase and subsequent hydrolysis to sphingosine by neutral ceramidase. Both ceramide and sphingosine can be absorbed into intestinal mucosal cells [102].

Interestingly, almost 20% of intestinal PLs are absorbed passively and without hydrolysation, and preferentially incorporated directly into HDL [43]. In addition, a substantial part of the dietary PL fraction is integrated into HDL particles already in the intestine that later join the plasma HDL pool. There is also evidence that PLs incorporated into lipoproteins of the blood stream, might be a more efficient delivery form than TAGs for PUFAs to several tissues and organs (i.e., brain, liver, lung, heart, etc.), including blood cells such as platelets and erythrocytes [43]. For example, piglets fed with a PUFA-TAG formula had a higher PUFA content in PLs bound to low-density lipoprotein (LDL, a lipoprotein for cholesterol transfer, derived from VLDL after its delivery/degradation of TAGs) than those fed with PUFA-PLs formula, while the opposite results were found in HDL PLs [103]. Thus, dietary PUFAs in form of TAGs or PLs affect the composition of PLs in HDL and LDL in different ways, and therefore the composition and functionality of lipoproteins and their distribution in the body and affect the fatty acid tissue incorporation in the host. The beneficial effects of PLs on blood and hepatic lipids have been studied in a number of animal experiments [104,105,106,107], and both cholesterol and TAG levels are affected upon treatment [104]. PLs have also been shown to increase levels of HDL in humans [108,109]. Many of the studies performed with PLs did not include PLs containing ω-3 PUFAs, indicating that PLs in general have beneficial effects [59,106,110]. However, it has also been shown that ω-3 PUFAs are better protected from oxidation when they are incorporated into PLs compared to TAGs. Other studies have demonstrated that PL-bound ω-3 PUFAs have more potent effects on blood plasma and liver lipid levels compared to PLs without ω-3 PUFAs [111,112]. In addition, dietary PLs are known to inhibit cholesterol absorption when added in significant amounts to the diet [113]. Several other mechanisms have been proposed for the effect of PLs on the reduction of cholesterol and other lipid absorption in intestine, such as their structure-related physical emulsifier properties and the ability to form a fat-water emulsion with cholesterol and other lipids, forming vesicles or micelles [114]. PLs play an important role during lipid intestinal absorption by facilitating the formation of micelles, while the cholesterol transport from the intestine into the enterocytes depends on the emulsification of the dietary fats with biliary secreted PLs, or with PLs from the diet. Intestinal PLs are also able to interact with the cellular membrane of enterocytes, reducing their cholesterol absorptive capacity [8].

It is also very interesting that the uptake of dietary PLs are mostly incorporated and affect the functionality of HDL-lipoproteins that have been characterised as the “good” cholesterol, because these lipoproteins not only remove excess cholesterol from blood stream and from atherosclerotic plaques, but also have exhibited anti-inflammatory and antioxidative properties. HDL also bares a plethora of cardioprotective enzymes such as PAF catabolic enzymes [115], contributing to the maintenance of endothelial cell homeostasis which protect the cardiovascular system [116].

During atherosclerosis and endothelial dysfunction, oxidation of lipoproteins also occurs, especially that of LDL that is transformed to oxidised-LDL (Ox-LDL), which migrates along with white blood cells to the subendothelial intima leading to the formation of foam cells and atherosclerotic lesions [23,28]. HDL and its enzymes seem to protect against these manifestations, while effort to increase HDL levels tends to be one of the main goals of dietary interventions and drug administration for cardioprotection. One of these HDL protective mechanisms involves the enzyme PAF acetyl-hydrolase (PAF-AH), which HDL bares. PAF-AH is a delicate Phospholipase A2 also referred to as Lp-PLA2 (lipoprotein associated Phospholipase A2) that protects against the production and activity of Ox-LDLs by promoting the catabolism of PAF and Oxidised-PLs (Ox-PLs) existing in Ox-LDL (especially those Ox-PLs that mimic PAF). Plasma-PAF-AH activity (both in LDL and HDL) is increased as a response to inflammation and oxidation, as a “signal terminator” [117]. However, during persistent LDL oxidation, PAF-AH is progressively inactivated (plasma-PAF-AH is incorporated mainly in LDL) and thus it loses its capacity to protect against the pro-inflammatory actions of PAF and oxidised-PLs mimicking PAF. On the other hand, dietary intake of PLs (especially those baring ω-3 PUFAs) increase HDL-levels and the incorporation of such anti-inflammatory and anti-oxidant dietary PLs to HDL, thus providing an additional protective mechanism by increasing plasma PAF-AH activity and by protecting the HDL-enzymes (such as PAF-AH) from oxidation-related inactivation [28]. The above is also in agreement with the beneficial *in vitro* and *in vivo* effects of several dietary PLs, which are shown in Table 1, especially on PAF-metabolism and HDL biofunctionality (including HDL-levels and increased PAF-AH activity) towards reduced PAF levels and cardioprotection.

## 2. Phospholipids of Animal Origin

Foods and fats of animal origin namely meat, eggs and dairy receive undue criticism from society and scientific communities due to their perceived negative effects on health upon consumption. Recent research trends have shown that these negative perceptions may be unwarranted as numerous research teams have shown that meat, eggs and dairy products, including some of their lipid fractions, may be associated with a positive effect on health when eaten in moderation despite their SFA and cholesterol content [23,114,118,119,120,121,122,123]. For the purpose of this review, Table 2 presents the phospholipid composition of a number of animal and marine species, however it is clear from the literature that the study of the phospholipid composition of many animal and marine food sources has been neglected as published research tends to focus solely on the fatty acid composition and not the phospholipid species composition.

### 2.1. Meat Phospholipids

Red and white meat contribute several important nutrients to the diet, including vitamins (B12 in particular), essential amino acids, iron, selenium, zinc, folic acids and fats. The phospholipid content of white meat from chicken and turkey is not well established in the literature. A study by Ferioli and Caboni [145] indicates that as with red meat, PC is the dominant species of phospholipid in raw chicken, followed by PE, SM, PI and PS. Similar findings were found for turkey meat (Table 2) [129]. For the purpose of this review, red meat is discussed in terms of their phospholipid content and anti-inflammatory activities.

The associated health benefits of red meat are controversial, and although contested there are clear indications that excess consumption of red meat and particularly processed meats may be associated with some forms of cancer and the development of CVDs [146,147]. Red meats (beef, veal, pork, lamb and mutton) are a rich source of phospholipids [125,148]; however, their compositions and structures are not well updated in the literature. The general phospholipid content of several meats is depicted in Table 2. The phospholipid content of beef, lamb and pork from mechanically deboned meat is reported to be 13.2%, 3.3% and 3.6%, respectively, of the total lipid content of the meat. In deboned beef, PC represents 56% of the total phospholipid content, followed by PE at 17%. Hamburgers or ground beef is consumed globally. In hamburgers, PC (53.4–57.2% phospholipid content) is the most abundant followed by PE (24%), with lesser quantities of PI (5.4–6.6%), SM (5.3–6.4%), CL (5.0–5.7%), and PS (1.9–3.7%) [126,149]. The phospholipids present in pork meat are found in similar quantities, where PC (58–63%) and PE (28–34%) are the most abundant followed by lesser quantities of PS and SM [128]. The total PUFA content of meat is generally low. Notably, the PUFA composition of PC in hamburgers is 29.8%, however the PUFA content of PE in hamburgers is 54.3%. The PUFA content in hamburgers is swelled by the enormous amount of arachidonic acid present (39.0%) [149]. This is of note as arachidonic acid is a ω-6 PUFA and is considered to possess pro-inflammatory properties and thus may contribute to CVD development. However, the abundant presence of PC in beef, which as highlighted by Lordan and Zabetakis [23] may be cardioprotective in nature, and may offset the inflammatory effects of the high arachidonic acid content of the meat. The arachidonic acid content also relates to the ω-6/ω-3 PUFA ratio, where a 1:1 ratio is considered ideal for a healthy lifestyle, however due to modern food production, the ratio is closer to 15:1 or even 17:1. This imbalance in the ω-6/ω-3 PUFA ratio is associated with the pathogenesis of several systemic inflammatory diseases such as obesity and CVDs [51,150].

There is also considerable concern that red meat consumption elevates levels of choline and L-carnitine. Phosphatidylcholine is broken down to choline, which is transformed by the intestinal microbiota to trimethylamine (TMA), which along with L-carnitine is metabolised to trimethylamine N-oxide (TMAO) [151]. It is thought that excess dietary phosphatidylcholine increases the levels of TMAO resulting in a pro-inflammatory and prothrombotic state leading to insulin resistance, type II diabetes, and cardiovascular disease [152,153]. However, research indicates that dietary choline may not be to blame, and that the presence of specific gut bacteria promotes the conversion of choline into TMAO [154,155]. Research has shown that dietary choline from phosphatidylcholine derivatives in dairy and marine sources possess anti-thrombotic properties, contrary to the effects of TMAO [80,95]. Further research is required to study the structures and composition of phospholipids of animal meat origin, in order to discern their biological effects upon consumption.

In addition, a variety of meats consumed as part of the Western diet, contain substantial amounts of ether-linked PLs, such as alkylacyl-sn-glycero-3-phosphocholine, choline and ethanolamine plasmalogens [156]. Interestingly, meat TAGs contain greater proportions of SFA than PLs, however ether-linked PLs generally contain more unsaturated FA than the usual and more abundant diacyl PLs. Such dietary ether-linked phospholipids could influence the lipid composition of host tissues to the extent that biological responses produced by ether lipid mediators would be affected. For example, the ingestion of ether-linked PLs may provide precursors for the production of either PAF or agonists of PAF (PAF-like molecules) [156].

### 2.2. Milk and Dairy Phospholipids

**Figure 2:**
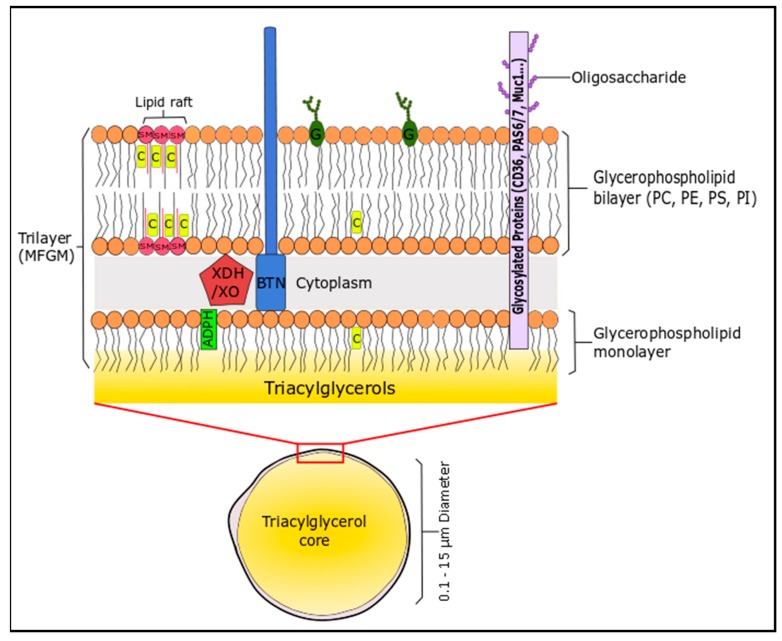
Illustration of the milk fat globule membrane. The sizes in this schematic are not in proportion. A phospholipid monolayer surrounds the triacylglycerol core, followed by a proteinaceous coat connecting the monolayer to the outer phospholipid bilayer. Adipophilin (ADPH) is located in the inner layer polar lipid layer, while xanthine dehydrogenase/oxidase (XDH/XO) is located between both layers. PE, PS and PI are generally concentrated on the inner surface of the membrane, whereas PC, SM, glycolipids (G), cerebrosides and gangliosides are mainly located in the external membrane. SM and cholesterol (C) can form rigid domains in the cellular membrane known as lipid rafts. Glycoproteins are distributed over the external membrane surface; these include butyrophilin (BTN), Mucin 1 (MUC1), PAS 6/7 and CD36.

The lipid profile of bovine milk is a complex mixture and can be distinguished by the fact that it is the most natural source of short-chain fatty acids (C4–C8, 4–13 wt % total FA), which are generally esterified on the *sn*-3 position of the triglyceride [157]. The non-polar (neutral) lipids (triglycerides or TG; 96–97% of milk lipids), the polar lipids (glycerophospholipids, sphingolipids, glycosphingolipids, glycolipids; 0.2–2% of milk lipids) and cholesterol create an oil in water emulsion to form milk. These lipids assemble into spherical milk fat globules of triacylglycerides (0.1–15 µm) that are engulfed in a complex trilaminar membrane (4–12 nm) composed of proteins, phospholipids and sphingolipids, suspended in an aqueous liquid phase, which is derived from mammary endothelial cells [158]. This unique structure is the milk fat globule membrane (MFGM), which consists of lipid (40%), proteins (60%) and cholesterol [159]. The membrane consists of phospholipids (mainly located on the outer leaflet) and cholesterol, which stabilises the TG-rich milk fat globule against coalescence and protects the core from lypolytic degradation and oxidation (Figure 2). Milk is a rich source of SFA, even though cow’s generally follow an unsaturated diet that includes PUFA, due to their presence in forage crops and seeds. The high levels of SFA are due to biohydrogenation of PUFA in the rumen of cattle [157].

The phospholipids present in bovine ovine and caprine milks are quantitatively minor constituents of milk lipids, however they possess beneficial techno-functional properties and are involved in various physiological processes and nutritionally valuable. Other sources of phospholipids in dairy products include MFGM fragments and lipoprotein particles, which are believed to be remnants of the mammary secretory cell membranes. PLs like the MFGM originate from the apical plasma membrane of the mammary gland secretory cell [2,3,158,159,160,161,162].

Although phospholipids only account for 0.32–1.0% of the total lipids of milk, they possess strong biological activity. The general phospholipid content of several milks is depicted in Table 2. The PL content of raw bovine milk is reported between 9.4 and 35.5 mg/100g. The phospholipid composition consists of PE (19.8–42.0%), PC (19.2–37.3%), PS (1.9–10.5%), and PI (0.6–11.8). The reported sphingolipid composition of raw milk consists of glucosylceramide (GluCer: 2.1–5.0%), lactosylceramide (LacCer: 2.8–6.7%) and SM (18.0–34.1%) [3]. The phospholipid content of small ruminants, such as ovine and caprine animals differs to that of bovine animals. Zancada, Pérez-Díez, Sánchez-Juanes, Alonso, García-Pardo and Hueso [131] reports the presence of 27.6 mg/100 g in caprine milk, and 29.8 mg/100 g in ovine milk. The phospholipid composition of ewes’ milk consists of PE (26.1–40.0%), PC (26.4–27.2%), PS (4.96–10.7%), PI (4.16–6.40%) and SM (22.6–29.7%), whereas, in goat milk, it has been reported as PE (19.9–41.4%), PC (27.2–31.9%), PS (3.2–14.0%), PI (4.00–9.37%) and SM (16.1–29.2%) [131]. It is well documented that the composition, structure, and properties of the fatty acids in milk are affected by several factors such as the breed, season, milking frequency, stage of lactation, nutritional status, and environmental conditions [163,164,165]. It has also been reported that these factors also affect the phospholipid content of milk [161,166,167,168,169,170,171]. As aforementioned, milk fat is characterised by short- and medium-chain fatty acids (C4–C14). These fatty acids are generally absent in the PL fraction of milk. PE tends to be highly unsaturated followed by PI and PC, whereas PC tends to be saturated compared to other glycerophospholipids. Sphingosine (d18:1) is the most prevalent sphingoid base in milk, that contains 18 carbon atoms, two hydroxyl groups, and one double bond. The fatty-acid pattern of SM is very uncommon with approximately 97% of the fatty acids were saturated, including C16:0, C18:0, C18:1n9, C22:0, C24:0 and C23:0. The latter accounts for over 17% of the fatty acid content of SM [3,172,173].

The peculiar fatty acid composition of SM allows the molecule to form in the cellular membranes and rigid domains with cholesterol, called lipid rafts, which are involved in different cellular processes [159]. The major sphingolipids in dairy products are GluCer, LacCer, and SM. Gangliosides are also present in dairy product in low concentrations (0.14–1.10 mg/100 mL) [174,175]. Lysophospholipids and PA are generally not present in dairy samples and occur due to the enzymatic activity of phospholipases [3]. Their origin is still unclear, but it is thought that could be formed because of hydrolysis occurring during milk processing or poor sample storage [2].

In terms of health benefits, milk polar lipids are now known to have several nutritional benefits. Sphingolipids and their metabolites including ceramide, sphingosine, and sphingosine phosphate have been found to be highly bioactive, having important effects on cell regulation and are linked to many inflammatory diseases [2,23,176,177,178]. Sphingolipids exhibit the ability to mediate intestinal inflammation and may prevent colon-related diseases including cancer [179,180,181,182,183]. Research has also indicated that the chemotherapeutic effects of dietary sphingolipids may also extend to other cancers such as breast and ovarian cancers [184,185]. Milk polar lipids in a high-fat diet fed to mice, did not induce white adipose tissue hypertrophy and inflammation but increased colonic goblet cells. These effects were attributed to the anti-inflammatory effects of the milk polar lipids, and in particular sphingomyelin derivatives [186]. Dairy SM has also been shown to reduce cholesterol and FA absorption through modulation of the cholesterol micellular solubility. The greater inhibitory effect of milk SM on lipid absorption appears to be associated with its greater saturation and longer chain-length of its fatty acyl group, which may allow for stronger hydrophobic interactions [187,188,189].

Recent research on dairy polar lipids has shown that PC derivatives in cheese [93] and yogurts [94,95] have strong antithrombotic and anti-inflammatory activities. In yogurts, cardioprotective PC derivatives have been isolated from yogurt polar lipids [95]. Sphingolipids, PE, and CL also tend to be highly bioactive in these dairy products [95]. Research has also shown that fermented dairy products have greater anti-inflammatory properties than unfermented milk [94,190]. Therefore, current research is focusing on the structural characterisation of these phospholipids and how they are biosynthesised during dairy product manufacture. Research suggests that these phospholipids may possess cardioprotective properties similar to phospholipids of marine sources, thus further research is warranted to assess the putative benefits of these lipids upon their consumption [23,190]. Overall, dairy product consumption seems to be associated with positive cardiovascular and metabolic health contrary to general perception [23,118,119,190,191,192,193,194,195,196,197,198], which may be due to the anti-inflammatory activities of dairy PLs [23].

### 2.3. Egg Phospholipids

Eggs are a valuable source of a wide variety of essential nutrients and bioactive compounds that can impact human health including high quality protein, fat-soluble vitamins, B vitamins, minerals, and choline, while providing relatively less saturated fat per gram compared to other animal protein sources. The egg yolk is one of the richest sources of dietary phospholipids. On average, one large egg contains 1.3 g of PLs by weight, which represents approximately the 28–30% of the total lipids of egg, while the remaining 66% are TAGs and 5% cholesterol. These lipids are almost exclusively found in the yolk, where PC is the predominant PL species accounting for approximately 72% of the total egg PLs. Other PLs are present in lesser quantities including PE (~20%), lyso-PC (3%), PI (2%), and SM (3%) (Table 2). The majority of egg PLs contain long-chain saturated and monounsaturated FAs, while the variety of the distribution of FAs can be somewhat reflective of the hen’s diet, age, and environmental conditions [8,114,123]. Because of their wide spectrum of activities, egg PLs have also been extensively used as pharmaceutical excipients for pharmaceutical formulations in oral, dermal, and parenteral products including liposomes [199].

Egg PLs are important contributors to the overall dietary PLs intake in the Western diet. Egg PLs contribute 10–40% (or 0.8 g) of daily consumed PLs in typical Westernised diet typical of the USA [114]. Egg PLs are highly bioavailable; PC is usually absorbed at approximately 90% efficiency. Consumption of eggs results in greater increases circulating HDL levels and enriches the HDL with PLs from egg. This is because dietary PLs are preferentially incorporated into plasma-HDL, where the beneficial effects are likely attributable to the consumption of egg PLs. It was also observed that the incorporation of TAGs in HDL was decreased [114,123,200]. In contrast to egg PLs, the absorption of egg-derived cholesterol is affected by the food matrix composition and can be altered by interactions with dietary PLs, potentially altering the mobilisation of cholesterol from micelles in the intestine [114]. Egg PLs reduce cholesterol and FAs absorption by possibly interfering with lipid mobilisation from mixed micelles. Although biliary PC is a critical emulsifier of dietary lipids and aids in their digestion and absorption in the GI tract, excess luminal PC appears to inhibit lipid absorption. Even though egg SM makes up only about 2% of total PLs in egg yolk, SM and other sphingolipids have also been shown to dose-dependently reduce the absorption of cholesterol, TAGs and FAs [114,187,201,202]. The influence of egg PLs on cholesterol absorption appears to be dependent on the FA saturation, (i.e., egg PC or saturated egg-PC inhibited the absorption of cholesterol into the lymphatic system greater than the more unsaturated soy PC) [114]. The reduced absorption of cholesterol, TAGs and FAs by dietary egg PLs appears to influence hepatic lipid levels and metabolism, while egg SM was also found to reduce the expression of hepatic nuclear receptors (such as peroxisome proliferator-activated receptor-α, PPAR-α), resulting in reduction of hepatic expression of genes involved in cholesterol biosynthesis and FA metabolism [114,203].

Egg PC and SM appear to regulate lipid absorption, hepatic lipid metabolism, and inflammation. In clinical studies, egg PL intake is associated with beneficial changes in serum biomarkers related to HDL function. In addition to the effects on lipid metabolism, dietary intake of egg PL may also reduce inflammation [114,200,204]. Consuming eggs for 12 weeks resulted in a reduction in plasma C-reactive protein (CRP) and an increase in adiponectin in overweight men; changes that were not observed with yolk-free egg substitute [204]. Egg consumption has also led to improvements in circulating plasma inflammatory markers in adults with metabolic syndrome [200]. The consumption of egg-derived unsaturated PLs is recommended especially in patients suffering from CVDs, since they modulate components of cell membranes, contribute to a decrease of cholesterol level and blood pressure [205]. In addition, hen egg yolk PLs were found to inhibit *in vitro* PAF-induced platelet aggregation, using cage-free hen egg yolk PLs. Thus, hen egg yolk was found to contain natural PAF-inhibitors that reinforce their nutritional value in terms of protection against CVDs [92].

The majority of research investigating inflammatory properties of PLs has focused on PC. For example, an *in vitro* study with hepatic cancer cell lines showed a dose dependent growth restraint when cancer cells were cultured in the presence of egg yolk PC (99% pure PC from egg yolk) and menaquinone-4 (vitamin K2). Furthermore, PC alone also showed a statistically significant reduction of cancer cells via death ligands (i.e., TNF-α), thereby promoting apoptosis by the activation of caspase-8 and -3, resulting in PAP (poly(A)-polymerase) inhibition [8,206]. However, as aforementioned, gut bacteria may be responsible for these observations.

However, even though egg-derived PLs have anti-inflammatory properties, they also seem to exhibit pro-inflammatory properties, via both direct and indirect mechanisms. For example, despite the evidence to suggest that PC is anti-inflammatory, egg intake has also been shown to dose-dependently increase post-prandial TMAO concentrations in plasma, and thus egg PLs have recently been implicated in the promotion of inflammation and atherosclerosis due to this increased formation of TMAO [123].

In addition, the existence of small amounts of the pro-inflammatory mediator PAF and its hydroxyl-PAF analogue (that mimics PAF-activities) was also found in the PCs fraction extracted by hen’s egg yolk [38]. A predictable finding, since PAF and its analogues are playing crucial roles in fertilisation [207]. Thus, even though the existence of small amounts of PAF and its analogues in hen’s egg-yolk seem to participate mainly in the reproductive system of hen, over consumption of eggs (much more than the dietary recommendations) may increase pro-inflammatory PL uptake (such as PAF and its analogues).

Given that numerous epidemiological studies have failed to conclusively find an association between egg intake and atherosclerosis, additional long-term studies are required to determine whether egg-induced TMAO production or pro-inflammatory PL uptake has detrimental effects on inflammation and disease risk. It is also crucial to discern whether the natural existence of PAF-inhibitors in egg-yolk and the perceived benefits of egg PLs intake on CVD risk markers outweigh any risk derived by potential TMAO formation and PAF uptake by over-consumption of eggs.

## 3. Marine Origin

### 3.1. Sources of Marine Phospholipids

Marine lipids can be derived from several fish, shellfish and algae, but also by Antarctic crustacean krill (*Euphausia superba*) and from marine industry by-products such as fish roe (fully ripe internal egg masses in the ovaries of fish) [43,208,209]. Fish contains between 1 and 1.5% PLs, while the amount of PLs in oil extracted from krill is typically around 40% of its total lipids [210,211]. PC derivatives are the predominant phospholipids present in salmon, tuna, rainbow trout, and mackerel; the second most abundant phospholipid is PE, PI, PS, lyso-PC, and sphingomyelin are also present in minor quantities [212] (Table 2). Fish roe from herring, salmon, pollock, and flying fish contain between 38 and 75% of their lipids in the form of PLs with PC being the predominant lipid class. Notably, the main PLs class of marine-derived PLs is PC, predominantly binding with unsaturated ω-3 PUFAs, with the most prevalent being EPA and DHA, but also stearidonic acid and docosapentaenoic acid (DPA). Marine organisms are enriched in these PUFAs by the aquatic food chain since the main source of ω-3 PUFAs are algae that can synthetize them de novo. Humans can only poorly synthesize ω-3 PUFAs from their precursor α-linolenic acid (ALA; 18:3ω-3) and thus the dietary intake of EPA and DHA is essential as they are extensively associated with optimal human health and protection against disease [43].

### 3.2. Oxidation of Marine Phospholipids—Pro-Inflammatory Mediators

ω-3 PUFAs such as DHA are highly susceptible to oxidation and the formation of toxic oxidized products such as aldehydes and hydroperoxides has been observed [213,214]. Furthermore, oxidized PLs with PAF-like structures that can mimic the inflammatory activities of PAF can be produced by the oxidation of marine PLs [215]. High amounts of oxidized products in the body over a prolonged period can cause oxidative stress, which can induce an inflammatory response [213]. PUFAs, EPA and DHA, need to be protected, and some different strategies have been used to avoid oxidation. Since the most stable ω-3 fatty acids are in the form of PLs, incorporation of ω-3 PUFAs into the PL structure increases their oxidative stability, suggesting that PLs baring PUFAs may be a more beneficial form of PUFAs than TAGs or esters [216]. For instance, it has been shown that DHA incorporated into PLs is more resistant to oxidation than both TAGs and ethyl ester bound DHA [217].

Marine PL products have revealed surprisingly high stability against oxidation [43]. There is speculation as to whether this is due to the natural content of antioxidants (e.g., astaxanthin) co-extracted with other lipids and PLs from the biomass, or if this is a function of the PLs themselves. Research suggests that both assumptions may be correct, due to the fact that other non-marine PLs, even when highly purified and devoid of antioxidants, are usually quite resistant to oxidation [58]. However, the oxidative stability of marine PLs is influenced by the quality, source, chemical composition of marine PLs and the degree of non-enzymatic browning reactions within marine PLs. In general, the non-enzymatic browning reactions in marine PLs are influenced by the marine PLs manufacturing processes. In addition, the use of marine PLs for food fortification is a challenge due to the complex nature of the degradation products that are formed during the handling and storage of marine PLs. Therefore, stabilisation of marine PLs in food systems with the addition of natural antioxidants should be further investigated [218]. For example, the combination of tocopherol, ascorbic acid, and lecithin has a higher protective effect on PUFAs than tocopherol, ascorbic acid, or lecithin individually [219]. To achieve the maximum protective effect, PUFAs such as DHA should be incorporated into PC or PE (one DHA molecule per lipid molecule) and both tocopherol and ascorbic acid should be added during food fortification or the manufacture of nutraceuticals or supplements [220]. Marine PLs products containing ω-3 PUFAs within their structure seem to provide resistance to oxidation PUFAs on their own.

### 3.3. Bioavailability and Biofunctionality of Marine Phospholipids

Consumption of ω-3 PUFAs, particularly the long-chain FAs EPA and DHA, has been reported to have beneficial physiological effects, including the reduction in the incidences of cardiovascular disease, cancer, diabetes, arthritis, and central nervous system disorders such as schizophrenia, depression, and Alzheimer’s disease [221,222]. Dietary ω-3 PUFAs have also exhibited beneficial effects in respect to essential FA deficiency in infancy (retinal and brain development), autoimmune disorders, Crohn’s disease, and cancers of breast, colon, and prostate [58]. The beneficial health effects of PUFAs have mostly been attributed to their anti-inflammatory and antithrombotic properties by their ability to decrease both the formation and tissue incorporation of ARA. This then prevents the overproduction of ARA-derived eicosanoids and reduces the release of inflammatory acute-phase proteins. By being precursors to lipid mediators (eicosanoids/docosanoids) or as ligands for transcription factors, these ω-3 PUFAs affect cell and tissue physiology and response to external signals. In addition, EPA and DHA can influence cell membrane fluidity, permeability or membrane protein-mediated responses. By these means, EPA and DHA have been proposed to support cardiovascular health as well as cognitive, visual, immune, and reproductive system functions [43]. There are also indications that they confer health benefits regarding tumorigenesis, hypertriglyceridemia, atherosclerosis, mental illness, dementia, bone health, and attention-deficit hyperactivity disorder (ADHD) [43]. Therefore, the development of products containing ω-3 EPA and DHA are of interest in nutraceutical development [223,224].

Currently, the global food and dietary supplement market for ω-3 PUFA (mostly EPA and DHA) is estimated to be 15,000–20,000 tons, derived from a total world production of fish oil of approximately 300,000 tons per year. However, the market for marine PLs is still in its infancy, even though research and development in this field has increased. This increased trend in using marine PLs has been attributed not only to their wide range of biofunctionalities but mostly because of their high bioavailability of ω-3 PUFAs (such as the EPA and DHA) that are mostly incorporated within the *sn*-2 position of the glycerol backbone of such PLs [43]. Other food sources for ω-3 PLs are very limited, and, as a result, the majority of ω-3 PL products are made from marine organisms [43]. 

Marine PLs are more efficient than marine TAGs in delivering ω-3 PUFAs to desired tissues [9,58]. With respect to plasma lipids and lipoproteins, fish oils are well known to decrease the levels of total cholesterol, blood TAGs content and LDL, while on the other hand increase HDL levels. However, decreasing the TAG levels and increasing the HDL levels in blood cannot be achieved by moderate intake of fish oil. Large amounts of fish oil administration are necessary for this purpose compared to marine PLs, since much lower quantities of marine PLs are required in order to achieve similar effects on decreasing the levels of plasma TAGs, total cholesterol and LDL but mostly in increasing HDL-levels, than the higher amounts of marine oils required (abundant in TAGs baring EPA or DHA).

However, it should be stressed that PLs by themselves, without the added benefit of ω-3 PUFAs, have exhibited several beneficial effects, such as to alleviate senescence [225,226], to modulate atherosclerotic plaques [9], benefit cognitive function [110], they possess anti-inflammatory activities [59,227,228], and they modulate blood and hepatic lipids (both cholesterol and TAG levels were reduced upon treatment while HDL levels were increased) in a number of animal experiments [104,105,106,107], and in humans [108,109]. All of the above studies with PLs that did not include ω-3 PUFAs containing PLs indicate that PLs in general have such beneficial effects. On the other hand, it was reported that PL-bound ω-3 PUFAs have more potent effects on blood plasma and liver lipid levels compared to PLs without ω-3 PUFAs [111,112], whereas ω-3 PUFAs are better protected from oxidation when they are incorporated into PLs (compared to TAGs), providing an additional beneficial biofunction of marine PLs concerning protection of PUFAs oxidation and any subsequently induced oxidative stress. An in depth presentation of the bioefficacy of ω-3 PUFAs marine PLs has been reviewed by Burri, Hoem, Banni and Berge [43].

### 3.4. Marine Phospholipids and Inflammation: The Missing Link

Since the early 1980s, extensive research concerning the anti-inflammatory properties of fish oils and marine products has been published. In the majority of these studies, most of the anti-inflammatory activities of fish oils were mainly attributed to the agonistic effects of ω-3 PUFAs (mostly EPA and DHA, which are abundant in marine products), towards ARA-based production of pro-inflammatory eicosanoids such as prostaglandins and leukotrienes [229,230,231], a mechanistic effect that is still emphasised to this day [8,43,232]. It is now well established that more complex mechanisms underlie the beneficial effects of fish consumption and administration of marine products that go far beyond the ω-3 PUFAs/ARA-related mechanism.

In addition, since the 1980s, mainstream studies on marine products have been based on using extracts of fish oil or purified ω-3 PUFAs without specifying the exact nature of these lipid mixtures. Poor sample preparation and lipid characterisation has led to studies using mixtures of neutral and polar lipids without being able to link the relevant bioactivities to specific lipid classes. Therefore, it is crucial to highlight that, in most of such studies, the administration of fish oil capsules or dietary intervention with fish was misinterpreted and characterised or identified as only an ω-3 fatty acids diet/supplement intervention. Thus, all the beneficial effects of fish and fish oil consumption were attributed to only the ω-3-PUFA constituents of fish and fish oils. However, fish and/or fish-oils do not only contain ω-3-PUFAs (esterified mostly in TAGs), but also PLs, which also contain ω-3-PUFAs in their structure. Other lipid constituents are also present in fish and fish oils that have different metabolic effects after absorption and far distinct biological activities, not only limited to their superior incorporation to plasma-lipoproteins and cell-membranes and bioavailability of their ω-fatty acids, but also to their reported anti-inflammatory activities through also other mechanisms than the ARA/eicosanoids pathway, such as the inhibition of the PAF-pathway and the modulation of PAF-metabolism [81,83] (see Table 1).

Interestingly, shark liver oils contain relatively low amounts of ω-3-PUFAs, however they have the ability to modulate the immune response through modification of PAF and diacylglycerol production, thus providing promising anti-cancer effects [233]. Furthermore, by using PAF-receptor-deficient knockout mice [234] exhibited that PAF/PAF-receptor linked signalling appears to be a prerequisite for the beneficial pro-inflammatory effects of fish oil based lipid infusions in murine models of acute inflammation-related lung injury [234]. In addition, marine products may also exhibit other anti-inflammatory mechanistic effects than that of ARA, as it was found that fish-oil supplementation in humans inhibited PAF-induced platelet aggregation, while ARA-induced platelet aggregation was unaffected by both fish-oil and/or olive oil supplementation in humans [235]. Thus, the anti-aggregatory effects of fish oil towards human platelets (and their subsequent anti-inflammatory properties) were attributed to inhibition of the PAF pathway and not that of ARA. What is more, ω-3 PUFAs on their own (and not fish-oil containing PUFAs) were not found to influence PAF-induced platelet aggregation, but only that of collagen-related platelet aggregation and thromboxane release in type II diabetic patients [236].

The discrepancy on the sample preparation has unfortunately led both the scientific community and the general public (i.e., industry nutritionists and consumers) to make a doubtful link between ω-3 PUFAs and inflammation-related disorders. This statement is supported by two recent systematic reviews and meta-analyses on the association between ω-3 fatty acid supplementation and risk and incidence of major CVD events [52,53]. Both studies concluded that insufficient evidence exists to suggest a beneficial effect of ω-3 PUFAs supplementation in adults with peripheral arterial disease regarding cardiovascular events and other serious clinical outcomes, whereas ω-3 PUFAs supplementation was not associated with a lower risk of all-cause mortality, cardiac death, sudden death, myocardial infarction, or stroke based on relative and absolute measures of association.

In addition, by using a meta-analysis, [54] have also investigated the efficacy of EPA and DHA supplements administration in the secondary prevention of CVD, and they have also found that there was a small reduction in cardiovascular death, which was disappeared when they excluded a study with major methodological problems, concluding that there is insufficient evidence of a secondary preventive effect of ω-3 PUFA supplements against overall cardiovascular events among patients with a history of cardiovascular disease.

Furthermore, in another recent systematic review of placebo-controlled randomised controlled trials (RCTs) of ω-3 PUFAs supplementation (that enrolled over 1000 patients with follow-up greater than one year) and meta-analysis of RCTs, carried out by Walz, Barry and Koshman [55], it was found that there is currently a lack of evidence to support the routine use of ω-3 PUFAs in the primary and secondary prevention of CVDs. It was also proposed by the authors of this study that pharmacists are ideally situated to engage patients in the discussion of the lack of benefit and possible risk of ω-3 PUFA supplements (since ω-3 PUFAs can increase the risk of bleeding and may interact with other medications that affect haemostasis, such as antiplatelet agents and warfarin) [55]. In addition, similar outcomes were also derived in another systematic review and meta-analysis on the association between fish consumption, long chain ω-3 fatty acids, and risk of cerebrovascular disease, carried out by Chowdhury, Stevens, Gorman, Pan, Warnakula, Chowdhury, Ward, Johnson, Crowe and Hu [56], where it was also proposed that the beneficial effect of fish intake on cerebrovascular risk is likely to be mediated through the interplay of a wide range of nutrients abundant in fish [56].

Therefore, we propose a different perspective: The beneficial effects of marine lipids are associated to the polar head of the phospholipid molecules and not only to the fatty acid moieties of the molecule (as it is outlined in Table 1).

Concerning the initial studies on the effects of fish oils towards platelet aggregation and the PAF-pathway [230,231,233,234,235,237], in 1996 Rementzis et al. reported the inhibitory activity of marine PLs from mackerel (*S. scombrus*) against PAF and thrombin induced aggregation of platelets [84]. Thus, for the first time providing evidence that the anti-PAF effects of fish oil towards platelet aggregation can be attributed to marine PLs and not to the ω-3 PUFAs. The most prominent proof that the anti-inflammatory and anti-thrombotic effects of fish and marine products (against the PAF-pathway) is mostly attributed at the marine PLs of fish was reported in 2000 by Panayiotou et al. where PLs extracts from fresh and fried cod (*Gadus morhua*) were found to exhibit potent inhibitory effect towards PAF-induced platelet aggregation, while this effect was related to protective effects of cod against atherosclerosis also through PAF-inhibition [86]. Since then, many studies have proposed a promising anti-inflammatory effect of marine PLs through targeting the PAF-pathway.

More specifically, Nasopoulou et al. have explored the anti-PAF and the anti-atherogenic properties of marine PLs extracted from wild and cultured sea bass (*Dicentrarchus labrax*) and gilthead sea bream (*Sparus aurata*) in several studies. These marine-PLs exhibited strong agonistic and antagonistic effect on PAF-induced platelet aggregation [81,82,238]. In addition, these marine-PLs inhibited the activities of the PAF-basic biosynthetic enzymes in human mesangial cells for the first time *in vitro* [87]. In an in vivo study the anti-atherogenic properties of these marine-PL extracts were further studied in hypercholesterolaemic rabbits [81]. HDL-C levels were significantly increased in the rabbits that were supplemented with marine-PLs in comparison with the control group. In addition, PAF-catabolic enzyme activity (Plasma PAF-acetylhydrolase) was significantly increased and platelet aggregation efficiency was reduced in these rabbits fed marine PLs in comparison to the control group. Finally, hypercholesterolaemic rabbits supplemented with marine-PLs developed early atherosclerosis lesions that were of a statistically significantly lower degree than that of the control group [81]. Furthermore, the basic PAF biosynthetic enzymes activities were reduced in the blood cells of the rabbits fed with marine-PLs, which resulted in reduced levels of PAF in their blood and reduced PAF activity, resulting in reduced formation of early atherosclerotic lesions. This was in contrast to the positive control group of rabbits that were not administrated marine-PLs [83].

In other studies, when cultured fish were fed with olive pomace as substitute for fish oil in fish feed, they exhibited satisfactory growth performance factors, statistically decreased levels of fatty acids, while also exhibiting potent biological activity against PAF-induced platelet aggregation, thus improving their cardioprotective properties [239]. It was also found that the most active lipid fractions of the fish were the polar in nature, mainly consisting of PLs and this was proved after extraction of polar lipids using counter count distribution to separate them for the neutral ones [240], and further HPLC-purification of these polar lipids [241].

**Figure 3:**
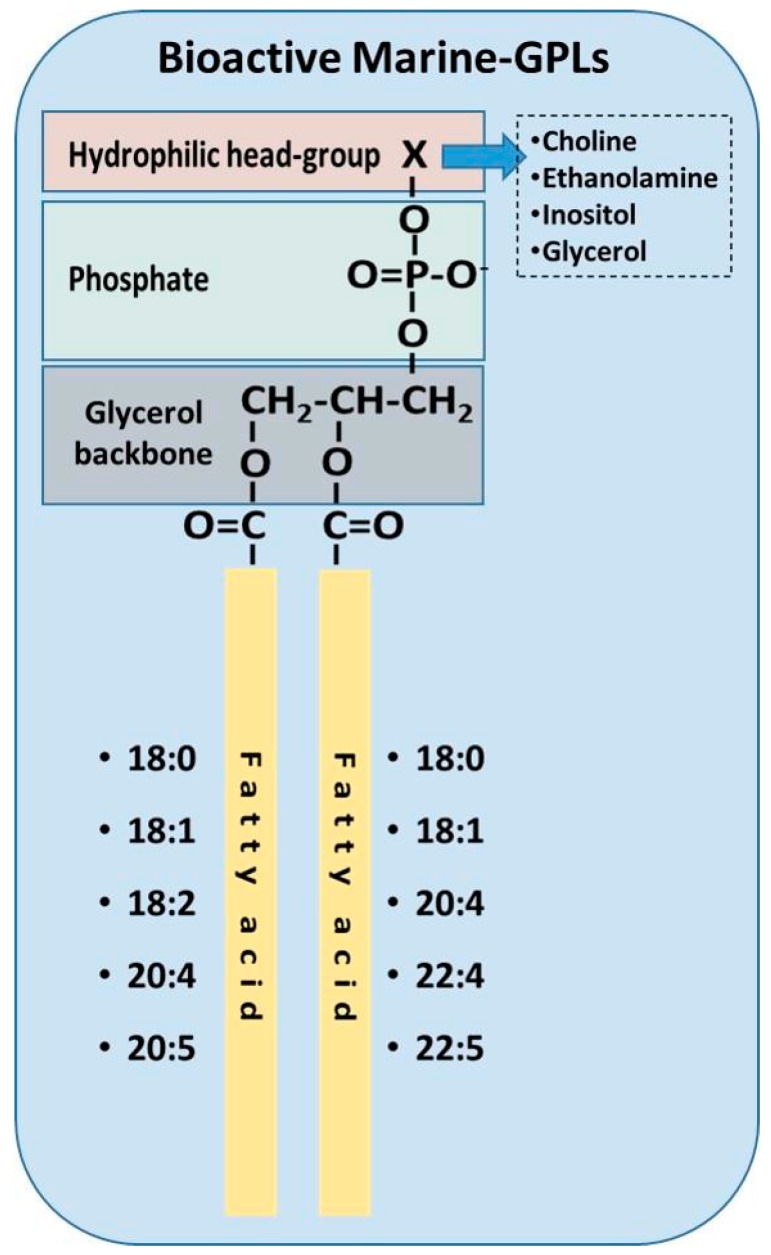
Structures of bioactive marine phospholipids as elucidated previously by Sioriki et al. and Nasopoulou et al. [80,85]. Generally, PC and PE derivatives exhibit the greatest bioactivity in marine sources.

Further analysis of these PLs from fish fed olive pomace was carried out using HPLC, GC-FID, GC-MS and LC-MS structural analysis. It was found that the marine PLs that possessed potent inhibitory effect towards PAF-induced platelet aggregation contained various diacyl-glycerophospholipids species, where the majority of them have either 18:0 or 18:1 fatty acids in the *sn*-1 position and either 22:6 or 20:2 fatty acids in the *sn*-2 position [80,85]. Furthermore, in the olive pomace fed fish, two PE-species were found to inhibit PAF-induced platelet aggregation *in vitro* [80]. The lipid structures of these novel bioactive PLs are summarised in Figure 3.

Thus, these studies have highlighted that, apart from their general health benefits, the administration of marine-PLs can modulate HDL-levels and functionality, but also they can modulate the levels, bioactivity and metabolism of inflammatory mediators such as PAF *in vitro* and *in vivo*, thus suggesting that these PLs may be protective against the onset and progression of atherosclerosis. Mechanistically, it is thought that the modification of PAF levels results in the reduction of platelet aggregation, inflammation and inflammatory manifestations such as atherosclerosis and CVDs. The appreciation of the role of HDL-functionality and PAF’s activity and metabolism in atherosclerosis provides a mechanistic framework for understanding and unravelling mechanisms where such bioactive food micronutrients as marine PLS are implicated in atherogenesis [83].

## 4. Conclusions and Future Perspectives

This review provides an overview of the existing literature concerning the active components of animal and marine PLs. Although PLs are generally considered a minor component of foods, PLs have the ability to interact with the cellular membranes, change their compositions and thereby influence a vast quantity of signalling and enzymatic processes. Therefore, many studies have reported the wide range of health benefits associated with PL consumption, without noticeable side effects. The main health effects of PL consumption include decreased absorption of cholesterol, increased plasma-HDL levels, better stability against oxidation (in comparison with TAGs) and modulation of inflammatory mediators (PAF and ARA); their levels, activities, and metabolism towards re-equilibrating the inflammatory-status and acquiring homeostasis, which can lead to reduced risk of inflammatory disorders such as atherosclerosis, CVDs, cancer, metabolic syndrome and diabetes, and renal disorders.

The beneficial effects of marine products towards these disorders are well documented. Previous perspectives have attributed most of these effects mainly to the pleiotropic activities of ω-3 PUFAs that are abundant in such sources. However, the results of very recent systemic meta-analyses have highlighted the lack of evidence to support the routine use of ω-3 PUFAs especially when administered as TAGs or esters, in the primary and secondary prevention of such disorders, since the beneficial effect of fish intake is likely to be mediated through the interplay of a wide range of nutrients abundant in fish. Thus, this review presents a new perspective, focusing on the well documented and promising beneficial effects of marine-PLs towards such inflammatory disorders, through a plethora of beneficial bioactivities. Furthermore, a higher uptake of such PLs might also be recommended for healthy individuals as a preventative strategy to improve public health. In addition, the existing negative perceptions associated with animal sources of dietary PLs, should also be re-evaluated and further studied, since recent research trends indicate that dairy PLs also possess anti-inflammatory properties with promising health effects, while both egg and meat PLs may contribute to the overall health benefits of PL consumption. Today, it has become clear that a balanced and healthy diet has positive effects towards preventing and managing inflammatory diseases. Thus, it is imperative to increase the awareness of the benefits of PL consumption.

PLs are a vital food manufacturing ingredient and are currently used as a food additive in a wide range of products including dairy products, instant drinks, baked goods, chocolate, and food supplements, to name a few. PLs are widely utilised as emulsification agents in the food manufacturing and pharmaceutical industry. The increased accessibility of high quality PLs derived from animal, egg, dairy and marine products and/or by-products will open the field of PL research to new opportunities, both for their future use as a superior nutritional source of bioactive molecules with beneficial effects, and for use in the food, pharmaceutical, nutraceutical and cosmetic industries. Further research and development in these areas has the potential to induce significant social, commercial, health, and environmental benefits.

## Figures and Tables

**Table 1:** Studies on the beneficial impact of PLs derived from food of the Mediterranean Diet towards inflammation-related disorders.

Studied Food and Components	Type of Study	Results
PLs of red and white wine, musts, grape-skins, and yeast	*In vitro* studies in washed rabbit platelets (WRPs) and in U937 macrophages *In vivo* postprandial dietary interventions studies in humans	Inhibition of platelet aggregation and modulation of PAF-metabolism towards reduced PAF-levels [73,74,75,76,77,78]
PLs of fish(Sea bass, sea bream, salmon, etc.)	*In vitro* studies in WRPs, human platelet rich plasma (hPRP) and in human mesangial cells (HMCs). *In vivo* studies in hyperlipidaemic rabbits	Inhibition of platelet aggregation, modulation of PAF-metabolism towards reduced PAF-levels and reduction of the thickness of atherosclerotic lesions in hypercholesterolaemic rabbits [79,80,81,82,83,84,85,86,87] Unpublished data for Salmon-PLs
PLs of olive oil and olive pomace	*In vitro* studies in WRPs and in HMCs. *In vivo* study in hyperlipidaemic rabbits	Inhibition of platelet aggregation and modulation of PAF-metabolism towards reduced PAF-levels and reduction of the thickness of atherosclerotic lesions in hypercholesterolaemic rabbits and regression of the already formed atherosclerotic lesions [87,88,89,90,91]
PLs of seed oils (soybean, corn, sunflower, and sesame oil)	*In vitro* studies in WRPs	Inhibition of platelet aggregation [88]
PLs of Hen egg	*In vitro* studies in WRPs	Inhibition of platelet aggregation [92]
PLs of dairy products (milk, yoghurt, cheese, etc.)	*In vitro* studies in WRPs and in hPRP	Inhibition of platelet aggregation [93,94,95] unpublished data for bovine, ovine and caprine milk, yogurt and cheese

**Table 2:** Typical composition of the phospholipid content in various foods of animal and marine origin.

PLs *	Total PLs ^1^	PC ^2^	PE ^2^	PI ^2^	PS ^2^	SM ^2^
**Egg**						
Egg yolk [8,113,114,123,124]	28–33	65–75	10–20	0.5–2.0	-	2–5
**Meat**						
Chicken Liver [113,124]	43–47	42–48	30–34	-	5–7	10–12
Chicken Breast [113,124]	67–70	48–52	23–25	-	12–14	7–9
Beef [113,124,125,126]	14–18	58–65	20–30	5–7	2–4	5–7
Pork [113,124,127,128,129]		55–63	20–34	-	1–8	1.2–6
Sheep-Lamb [129]	42	38–55	25–31	-	-	4–7
Rabbit [127,129]	23	51–65	20–24	4	4–8	-
Pigeon [127,129]	28–66	33–49	26–46	2–8	3–5	3–5
Duck (muscle) [130]	30–45	25–30	5–10	trace	trace	1–2
Turkey [129]	33–80	38–60	30–42	-	-	2–7
**Dairy Products**						
Cow’s Milk [3,8,113,124]	0.3–1.1	20–40	20–42	0.6–12	2–11	18–35
Ewes’ milk [131]	0.2–1.0	26–28	26–40	4–7	4–11	22–30
Goat milk [131]	0.2–1.0	27–32	20–42	4–10	3–14	16–30
**Marine Products**						
General Marine Composition [8,113,124,132,133,134]	2–95	45–90	5–35	1–6	1–11	1–15
Squid [113,124]	64–67	70–75	8–12		6–8	7–11
Cod [113,124,129]	24–30	50–77	12–25	3–4	4–6	5–11
Salmon roe [135]	30	80	13	4	trace	3
Salmon [129,136]	45–50	50–62	10–40	5–7	1–7	0.2–1
Gilthead Sea Bream (muscle) [137,138]	1–5	45–60	20–30	5–8	3–4	2–5
Sea Bass (muscle) [139]		62	20	7	4	3.4
Sea Bass (egg) [140]	10–22	11–15	12–14	47–66	-	5–18
Trout (muscle) [127,129]	12–19	66	21–25	2	4	2
Surgeonfish (muscle) [141]	9	56	29	7	4	-
Grouper [142]		29–48	4–13	10–18	2–4	11–14
Black Rockfish [143]	3–20	30–60	20–40	trace	trace	trace
Molluscs [144]		35–50	21–37	4–6	5–12	5–17

* Various foods are given on the left column with the relevant references. The table contains the typical composition of the referred PLs, which may differ depending on its source and the analytical method employed. ^1^ Mean values expressed as % of total lipid composition. ^2^ Expressed as % of total phospholipids. Abbreviations: PLs, phospholipids; PC, phosphatidylcholine; PE, phosphatidylethanolamine; PI, phosphatidylinositol; PS, phosphatidylserine; SM, sphingomyelin.
